# Neural Organization of the Optic Lobe Changes Steadily from Late Embryonic Stage to Adulthood in Cuttlefish *Sepia pharaonis*

**DOI:** 10.3389/fphys.2017.00538

**Published:** 2017-07-27

**Authors:** Yung-Chieh Liu, Tsung-Han Liu, Chia-Hao Su, Chuan-Chin Chiao

**Affiliations:** ^1^Institute of Systems Neuroscience, National Tsing Hua University Hsinchu, Taiwan; ^2^Department of Life Science, National Tsing Hua University Hsinchu, Taiwan; ^3^Institute of Molecular Medicine, National Tsing Hua University Hsinchu, Taiwan; ^4^Institute for Translational Research in Biomedicine, Kaohsiung Chang Gung Memorial Hospital Kaohsiung, Taiwan

**Keywords:** ontogenetic development, neural connections, cell islands, visuomotor control, visual lateralization, cephalopods

## Abstract

The optic lobe is the largest structure in the cuttlefish brain. While the general morphology of the optic lobe in adult cuttlefish has been well described, the 3D structure and ontogenetic development of its neural organization have not been characterized. To correlate observed behavioral changes within the brain structure along the development of this animal, optic lobes from the late embryonic stage to adulthood were examined systematically in the present study. The MRI scan revealed that the so called “cell islands” in the medulla of the cephalopod's optic lobe (Young, 1962, 1974) are in fact a contiguous tree-like structure. Quantification of the neural organizational development of optic lobes showed that structural features of the cortex and radial column zone were established earlier than those of the tangential zone during embryonic and post-hatching stages. Within the cell islands, the density of nuclei was decreased while the size of nuclei was increased during the development. Furthermore, the visual processing area in the optic lobe showed a significant variation in lateralization during embryonic and juvenile stages. Our observation of a continuous increase in neural fibers and nucleus size in the tangential zone of the optic lobe from late embryonic stage to adulthood indicates that the neural organization of the optic lobe is modified along the development of cuttlefish. These findings thus support that the ontogenetic change of the optic lobe is responsible for their continuously increased complexity in body patterning and visuomotor behaviors.

## Introduction

Cephalopods have the most sophisticated central nervous system (CNS) among all invertebrates (Nixon and Young, 2003). These complex brain structures reflect their intricate behaviors (Hanlon and Messenger, 1996). Within their CNS, a pair of optic lobes takes up about two-thirds of the total brain mass and these are known to have functions in visual processing and visuomotor control (Boycott, 1961; Young, 1962, 1974). Characterizing the neural structure of the optic lobes is thus essential for our understanding of the neural basis of cephalopod behavior.

**Figure 1:**
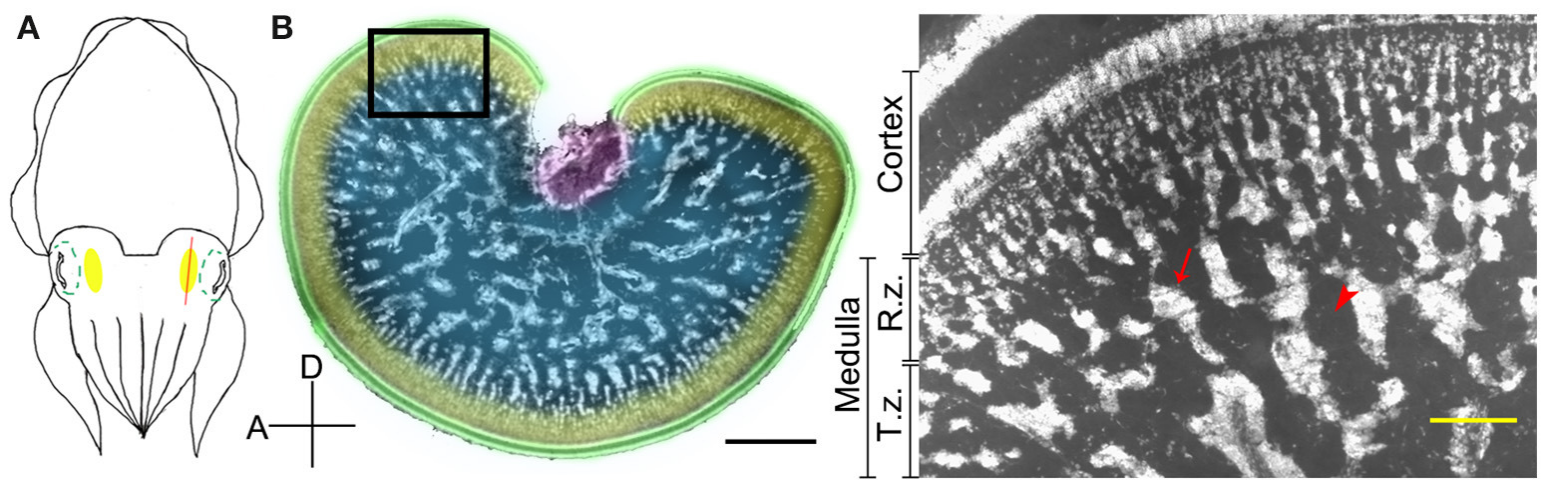
The optic lobe of the cuttlefish, *Sepia pharaonis*. **(A)** A schematic diagram showing the position of the optic lobe (yellow area) relative to the eye (green dashed outlines) in a top view of the cuttlefish. The red line depicts the plane of the cross section. **(B)** A cross section of the optic lobe showing the general morphology. This cryosection of the left optic lobe of a cuttlefish (ML = 22.5 cm) was counterstained with a nuclear dye to visualize the cell organization. Green, cortex; Yellow, radial column zone (R.z.); Blue, tangential zone (T.z.); Purple, optic tract region. Inset, a high magnification image of the optic lobe from the same location of a different cuttlefish (ML = 8 cm) showing the various distinct zones. Arrow, cell islands; Arrow head, an area occupied mostly by neural fibers. D, dorsal; A, anterior. Scale bar, 2 mm (Inset, 200 μm).

The optic lobe in cephalopods is a kidney-shaped brain structure located behind the eye ball (Figure 1A). It can be divided into two parts, the outer cortex and the central medulla (Boycott, 1961; Figure 1B). The cortex, also called the deep retina (Cajal, 1917), receives visual signals directly from the retina. It consists of two cell-rich granular layers with a single fiber-rich plexiform zone in-between (Young, 1962, 1974). It covers most of the optic lobe surface except for the optic tract region. In contrast, the medulla can be separated into two regions, the outer radial column zone and the central tangential zone (Young, 1962, 1974). The radial column zone, which contains numerous columnar structures of stacked cells and radially arranged neural fibers, lies beneath the outer cortex. When neural fibers from the radial column zone extend deeper into the center of the medulla, most of these fibers become tangentially arranged, thus this area is called the tangential zone (Young, 1974). Cell bodies within the tangential zone are clumped together into characteristic “cell islands” that are surrounded by neuropil, and there is no obvious histological differentiation in this region (Boycott, 1961; Young, 1962, 1974). Furthermore, it has been confirmed using phalloidin and alpha-tubulin staining that the space other than cell islands in the tangential zone are fully occupied by neural fibers in pygmy squids (Shigeno and Yamamoto, 2002; Wollesen et al., 2009). In cuttlefish, direct electrical stimulation of the cortex results in no obvious behavioral change, but stimulating the medulla evokes a range of body patterns unilaterally or bilaterally (Boycott, 1961). In addition, electrical stimulation of the medulla also produced various types of locomotive behavior (Chichery and Chanelet, 1976, 1978). These early experiments suggest that the cortex is responsible for visual information processing and the medulla is the motor command center for dynamic body patterning (Messenger, 2001). Despite our overall understanding of optic lobe structure and function, the detailed neural organization and the mechanisms underlying its control of body pattern generation have not been fully characterized.

“Live fast and die young” is an aphorism that well describes most modern cephalopods (O'Dor and Webber, 1986). Various lines of evidence indicate that most cephalopods complete their life cycles in one to two years (Boyle, 1983). This life style suggests that their brains must develop rapidly to meet their behavioral needs. Thus, characterizing the ontogenetic changes within the optic lobe in cephalopods and comparing with other fast growing animals may provide insights into the evolution of neural adaptation. Furthermore, it is known that early visual experience in embryos or hatchlings is important for the development of various visual behaviors (Dickel et al., 2000; Poirier et al., 2005; Darmaillacq et al., 2006; Lee et al., 2010, 2012; Guibe and Dickel, 2011; Romagny et al., 2012). Thus, examining the optic lobe structure from late embryonic to juvenile stages may also shed light on the neural plasticity of these observed behavioral adaptations. Earlier ontogenetic studies of brain structure in other cephalopod species are informative as they have helped to elucidate the growth pattern of the optic lobe (Meister, 1972; Marquis, 1989). In sepiolid squids, it has been found that both the volume of the optic lobe and its proportion in the brain keep increasing throughout the animal's embryonic stages (Kerbl et al., 2013). In oegopsid, lolignid, and pygmy squids, structural studies also revealed that, in the optic lobe, neuropil appear earlier in the cortex and tangential zone than in the radial column zone (Shigeno et al., 2001b,c; Yamamoto et al., 2003). Furthermore, it has been shown that the tangential zone of oegopsid and lolignid squids undergo a significant morphological change as they move from the embryonic to the juvenile stage (Shigeno et al., 2001a; Kobayashi et al., 2013). Despite the success of these early descriptive studies in squids, a systematic and quantitative study of the optic lobe from embryonic stage to adulthood in cuttlefish is crucial for correlating the observed behavioral plasticity with the optic lobe structure at various developmental stages of the animal.

In addition, behavioral lateralization was recently reported in the cuttlefish wherein the animals show side-turning preferences in a T-shape apparatus (Jozet-Alves et al., 2012b). This observed visual lateralization is task and age dependent in juvenile cuttlefish. It has been suggested that the turning bias in cuttlefish results from an eye use preference. Further analysis has revealed that there is an individual variation in the magnitude of the optic lobe asymmetry (Jozet-Alves et al., 2012a). Although the cerebral correlates of visual lateralization are apparent, it is important to further compare the internal structures of the left and right sides of the optic lobe in developing cuttlefish and to determine the structural basis of this observed visual behavioral asymmetry.

In the present study, the optic lobes of the pharaoh cuttlefish *Sepia pharaonis* were collected at different time points from late embryonic stage to adulthood. An MRI scan was used to reconstruct the internal structure of the optic lobe in an adult cuttlefish and the so called “cell islands” in the medulla of cephalopod's optic lobe were found to be contiguous. Histological examination of the optic lobes confirmed that the morphological features of the cortex and radial column zone were established earlier than those of the tangential zone during the embryonic and post-hatching stages, and neural fibers and cellular organization in the tangential zone increased and modified along the development of the cuttlefish. Furthermore, comparing the internal structures of the left and right optic lobes revealed that lateralization is evident in the cortex and radial column zone during the embryonic and juvenile stages. These morphological observations are discussed with respect to the behavioral development of the cuttlefish and adaptation by the cuttlefish.

## Materials and methods

### Animals

Embryonic and early post-hatching cuttlefish, *S. pharaonis*, were reared from eggs collected at Keelung, Taiwan. All eggs were transported to the National Tsing Hua University and maintained in the laboratory using two closed-circulation aquarium systems (700 L each; water temperature approximately 24°C). The room was kept on a 12 h light and 12 h dark cycle. Sub-adult and adult cuttlefish (sex undetermined) were obtained from local fishermen at Keelung, Taiwan. For embryonic and juvenile cuttlefish, the optic lobes were collected when they attained an appropriate stage or the required mantle length (Table S1). The embryonic stages were determined based on developmental characterizations of *S. pharaonis* (Lee et al., 2016) and *S. officinalis* (Lemaire, 1970). The post-hatching stages (juvenile, sub-adult, and adult) were determined based on developmental processes recorded in a previous *S. pharaonis* culture study (Minton et al., 2001). In a separate experiment, additional optic lobes from cuttlefish of embryonic stage 24 (*N* = 6), mantle length 2.8 cm (*N* = 2), 4 cm (*N* = 2), 17.7 cm (*N* = 2), and 19 cm (*N* = 2) were used for immunostaining studies (see below). These samples were also included in the analysis of cell size in the optic lobe.

### Histology

All animals were anesthetized using 3% MgCl_2_ added to sea water (Mooney et al., 2010). Each pair of optic lobes located behind the eyes (Figure 1A) was carefully dissected out and fixed immediately using 10% formalin in sea water for at least 3 days. The samples were then placed in 70% ethanol for storage. A day before cryosectioning, the optic lobes were incubated with a mixture of OCT (tissue freezing medium) and 30% sucrose solution. Immediately before sectioning, the samples were embedded in OCT and placed on the stage of a cryostat (CM3050S, Leica). A series of 30 μm slices was cut along the sagittal plane from the lateral side to the medial side of the optic lobe (Figure 1). To visualize the internal structure of the optic lobe consistently, only the middle section (50% of sections from the lateral side) was collected, unless stated otherwise. In a separate experiment, 10 μm slices were collected for the immunostaining study. The optic lobe slices were rinsed using PBS (phosphate-buffered saline), and then stained with a nuclear dye, either DAPI (4′,6-diamidino-2-phenylindole) or PI (propidium iodide), to visualize the cell organization (Table S1). In the immunostaining experiments, the optic lobe slices were incubated with 10% normal donkey serum, 0.5% Triton X-100, and 0.1% sodium azide in PBS for 1 h at room temperature. After blocking, the slices were incubated with the primary antibody against acetyl α-tubulin (dilution 1:200; T7451, Sigma) for 1 day at 4°C to label neural fibers (Klagges et al., 1996; Shigeno and Yamamoto, 2002). After extensive rinsing with PBS, the secondary antibody, donkey anti-mouse IgG conjugated with DyLight fluorophore 488 (dilution 1:250; Jackson), was applied overnight at 4°C to visualize the immunoreactivity. To ensure the specificity of the primary antibodies, a control experiment of only the secondary antibody without the primary antibody was conducted. The results confirmed that neural fibers in the optic lobe can be labeled only when the primary antibodies were applied (data not shown). Finally, after rinsing with PBS, the samples were mounted with glycerol mounting medium for fluorescent imaging.

### Image acquisition

Histological and immunostaining images of the optic lobe slices were acquired on an upright fluorescent microscope (Axioskop 2 mot plus, Zeiss) using either a 5X (A-Plan, 0.12 NA, Zeiss) or a 10X (Plan-Neofluor, 0.3 NA; Zeiss) objective lens depending on the sample size, or on a fluorescent dissecting microscope (Stemi SV11, Zeiss). The high resolution fluorescent images of showing nuclei and neuropil were acquired on a confocal microscope (LSM 510, Zeiss) using a 40X objective lens (Plan-NEOFLUAR, NA 0.75, Zeiss). In addition, the left optic lobe of a sub-adult cuttlefish *S. pharaonis* (ML = 16 cm) was subjected to the MRI scanning at the Kaohsiung Chang Gung Memorial Hospital (9.4T, Bruker BioSpec 94/20 USR) to obtain its 3D structure. Before scanning, the sample was embedded in agar containing ferric ions to reduce background noise. The MRI scanning system is made up of a self-shielded magnet with a 20 cm clear bore and a BGA-12S gradient insert (12 cm inner diameter) that offered a maximal gradient strength of 675 mT m^−1^ and a minimum slew rate of 4,673 Tm^−1^s^−1^. The optic lobe was imaged at high resolution with TurboRARE-3D-torun sequence (TR/TE = 3,000/48 ms, NEX = 2). The stack of MRI data was then processed to make a movie of the stereo image of the optic lobe.

### Image analysis

Quantifications of neural organization from the histological images of the optic lobe slices were done using ImageJ (National Institutes of Health, USA). The thicknesses of the cortex and of the radial column zone were measured separately (Figure 1B). The width of the radial column was determined by measuring the lateral spread of the columnar-like stacked nuclei, and the density of the radial columns was obtained by counting the number of radial columns along the circumference of the medulla in the optic lobe. The cross-sectional areas of the optic lobe, medulla, tangential zone, and cell islands were also determined accordingly (Figure 1B). Since all measurements from the left and right optic lobes were monotonically related (assessed by the Spearman's rank correlation coefficients; Table S2), the data from both left and right optic lobes were combined during analysis. However, to assess the optic lobe lateralization of individual cuttlefish, the measurements from the left and right sides were compared at each developmental stage.

To quantify the complexity of the cell islands in the MRI scan, the tree-like structure (see below) was analyzed by measuring the shortest distance of each branch point from the optic tract region. To examine the proportions of neural fibers in the optic lobes of cuttlefish at different ontogenetic stages, fluorescent signals of the immunostaining images were first thresholded and the areas were then measured. To quantify the cell size and density in the cell islands, the tangential zone was divided into two groups, peripheral and central areas, except for the optic lobe of embryonic stages where the cell islands are relatively homogeneous. The average nucleus size in the cell islands was determined for cuttlefish at each different ontogenetic stage by measuring the areas of randomly selected individual nuclei (*N* = 30) in the peripheral and central regions separately. Similarly, the average nucleus density in the cell islands was determined by counting the number of nuclei within three randomly assigned ROIs in the peripheral and central islands separately. The average nucleus size in the cortex and the radial column zone was also determined using the similar approach. For statistical analysis, the one-way ANOVA and *post hoc* Tukey's test were used to determine the significant difference after assessing the data normality with the Shapiro-Wilk test (SigmaPlot, NA).

## Results

### Cell islands are contiguous throughout the medulla of the optic lobe

**Figure 2:**
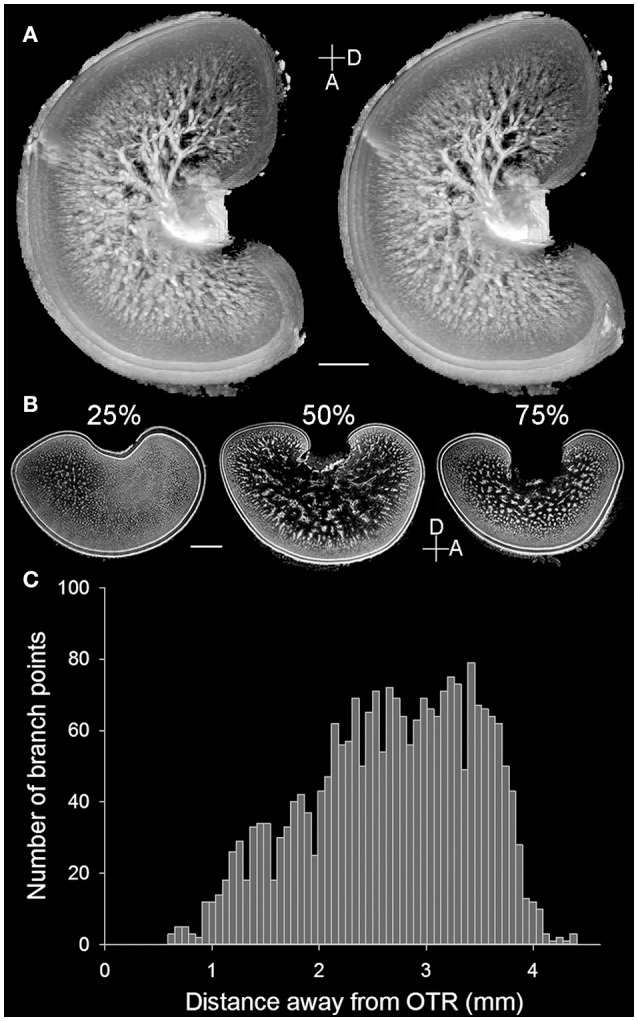
Cell islands in the medulla of the optic lobe are a continuous structure. **(A)** A pair of stereo images enhanced and reconstructed from the MRI scan showing the 3D structure of cell islands (light areas) in the medulla of the left optic lobe of a cuttlefish (ML = 16 cm). Note that the small opening on the left side of the optic lobe was an artifact caused by the electrode penetration from a separate study. **(B)** Three cross cryosections at different percentages from the lateral to the medial side of the right optic lobe of a cuttlefish (ML = 19.5 cm) showing the distinct morphologies of the cell islands. D, dorsal; A, anterior. Scale bar, 2 mm. **(C)** The branch point numbers of the 3D structure of cell islands shown in **(A)** increase steadily as the distance from the optic tract region (OTR) increases.

The present study showed the detailed neural organization in the optic lobe of the pharaoh cuttlefish *S. pharaonis*, which is consistent with the early Cajal staining study of functional brain organization of the common European cuttlefish *Sepia officinalis* (Boycott, 1961). However, the MRI scan of the optic lobe from a sub-adult *S. pharaonis* revealed for the first time that the so called “cell islands” present in the medulla (Young, 1962, 1974) are contiguous and have a tree-like structure (Figure 2A; see the Movie S1 in the Supplementary Information). A careful examination of these cell islands showed that this tree-like structure spread out from the optic tract region (OTR) with a continuously increasing number of branch points until reaching the radial column zone (Figure 2C).

Detailed examination of the histological images of the optic lobe slices showed that the cellular organization and fine structures are conserved throughout the optic lobe, except for the optic tract region (the main output region of the optic lobe) which is located closer to the medial and dorsal sides. Furthermore, the radial column zone (the main input region which receives visual signals from the eyes) is slightly thicker on the lateral and ventral sides of the optic lobe. As a result, the tangential zone could hardly be observed in the sagittal section of the optic lobe at 25% from the lateral side (Figure 2B, left), and the boundary between the radial column zone and the tangential zone were difficult to discern in the section at 75% from the lateral side (Figure 2B, right). Since the middle section of the optic lobe slices (50% from the lateral side) exhibits the general characteristics of the optic lobe morphology most clearly, such as the cortex, radial column zone, and tangential zone (Figure 1B), which represents a canonical view or a vantage point of the optic lobe, only the middle sections were used to examine the ontogenetic changes that occur during the development of the neural organization of the optic lobe.

### Features of the cortex and radial column zone are established earlier than those of the tangential zone during embryonic development

**Figure 3:**
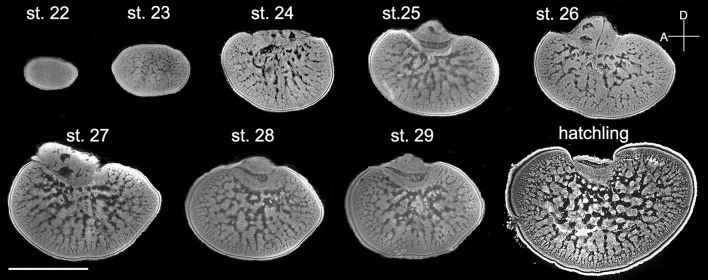
The distinct structures of the optic lobes gradually appear during the late embryonic stages. Middle sections of the left optic lobes from different embryonic stages of cuttlefish. Nuclear staining was used to visualize the cell organization in the optic lobe. D, dorsal; A, anterior. Scale bar, 1 mm.

Since the retina of cuttlefish embryos becomes reddish and the optic lobes become enlarged at stage 22 (Lee et al., 2016), the morphological development of the optic lobe was examined from this stage onward (Figure 3). At stage 22, the entire optic lobe was filled with cells and there was hardly any space for neuropil. The boundary between the cortex and the medulla was less distinct. Similarly, the boundary between the radial column zone and tangential zone was unrecognizable at this stage. At stage 23, although the characteristics of the radial column zone were still missing, space for neuropil appeared in the center of the medulla. The boundary between the cortex and the medulla was discernible and the two granular layers of the cortex were distinct. At stage 24, the feature of the radial column zone first appeared and the tangential zone had even more space for neuropil. The size of the optic lobe was also found to have enlarged significantly. The basic structures of the optic lobe were established at this embryonic stage. From stage 25 to stage 29, while the size of the optic lobe only increased moderately, the space for neuropil in the tangential zone continuously expanded. Note that the optic lobe in stage 27 appeared to be slightly larger than those in stages 28 and 29 due to individual size differences (Figure 3). Nevertheless, this observation supports that the size of the optic lobe does not increase significantly at late embryonic stages. At hatching (or stage 30), the size of the optic lobe was much larger than during previous embryonic stages, and the boundaries between the cortex and medulla, as well as between the radial column zone and tangential zone were much more distinct when compared with the embryos.

**Figure 4:**
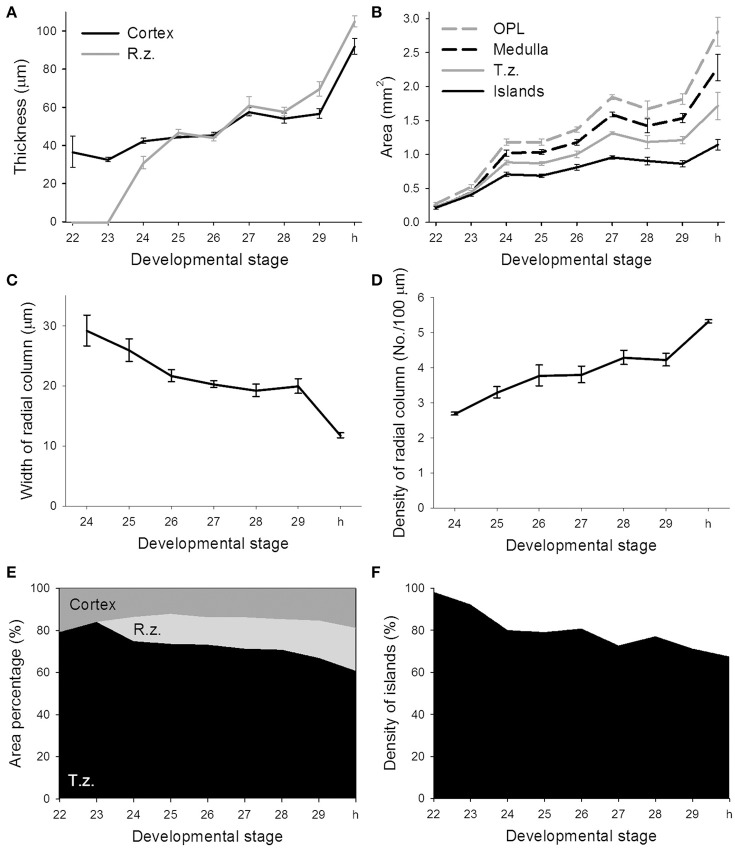
The features of the radial column zone first appear at stage 24, while the proportion of cell islands decreases throughout the embryonic development of the optic lobe. **(A)** The thickness of the cortex and radial column zone (R.z.) of the optic lobe steadily increases during embryonic development. **(B)** Various areas of the optic lobe (OPL), medulla, tangential zone (T.z.), and cell islands gradually increase throughout embryonic development. **(C)** The width of the radial column steadily decreases during embryonic development. **(D)** Density of the radial columns gradually increases throughout embryonic development. **(E)** The proportions of the cortex, radial column zone, and tangential zone within the optic lobe change during embryonic development. **(F)** Density of cell islands in the tangential zone of the optic lobe decreases as the embryo develops. h, hatchling.

To quantify morphological changes of neural organization during the development of the optic lobe, the thickness of the cortex and radial column zone was measured from stage 22 to hatching (Figure 4A). It is apparent that the cortex increased steadily during embryonic development except from stage 29 to hatching, but the radial column zone was not recognized until stage 24 and showed a steeper increase in size throughout the latter embryonic stages. A careful examination of the organization of the radial column zone showed that the width of radial columns decreased significantly throughout the late embryonic development (Figure 4C). As a consequence, the density of radial columns increased steadily from stage 24 to hatching (Figure 4D). When the areas of the optic lobe, medulla, tangential zone, and cell islands were compared, the results confirmed previous observations that the size of the optic lobe showed two distinct fast growing periods, from stage 23 to stage 24 and from stage 29 to hatching (Figure 4B). While the areas of the medulla and tangential zone followed a similar growing pattern to that of the optic lobe, the area made up of the cell islands increased relatively slowly. As a consequence, the proportion of the cortex and medulla in the optic lobe remained moderately stable throughout embryonic development, but the radial column zone took up a significant space from stage 24 onward and the tangential zone became relatively smaller as the embryos grew (Figure 4E and Table S3). Since the increase in the cell islands was slower than that of the tangential zone, this resulted in the density of cell islands in the tangential zone of the optic lobe decreasing continuously throughout embryonic development (Figure 4F and Table S3).

### Neural fibers in the tangential zone increase continuously from post-hatching to adulthood

**Figure 5:**
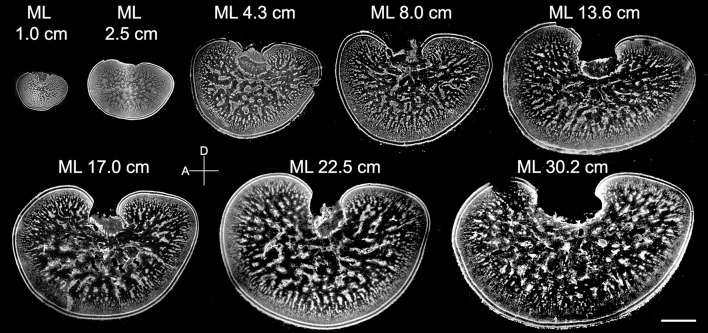
The neural organization within the medulla of the optic lobe changes continuously from hatching to adulthood. Middle sections of the left optic lobes from different mantle lengths (ML) of cuttlefish. Nuclear staining was used to visualize the cell organization in the optic lobe. D, dorsal; A, anterior. Scale bar, 2 mm.

Although the general morphology of the optic lobe from different sizes of cuttlefish was similar to the one observed in late embryos, the proportion of neuropil in the tangential zone was found to transform continuously throughout the entire post-hatching life (Figure 5). Specifically, much of the size increase of the optic lobe was a result of an expansion of the medulla rather than the cortex, and much of the area expansion of the medulla at adulthood was due to the growth of the tangential zone rather than the radial column zone. Furthermore, the space for neuropil in the tangential zone enlarged steadily, indicating that there was a continuous increase in neural fibers among the cell islands as cuttlefish grew.

**Figure 6:**
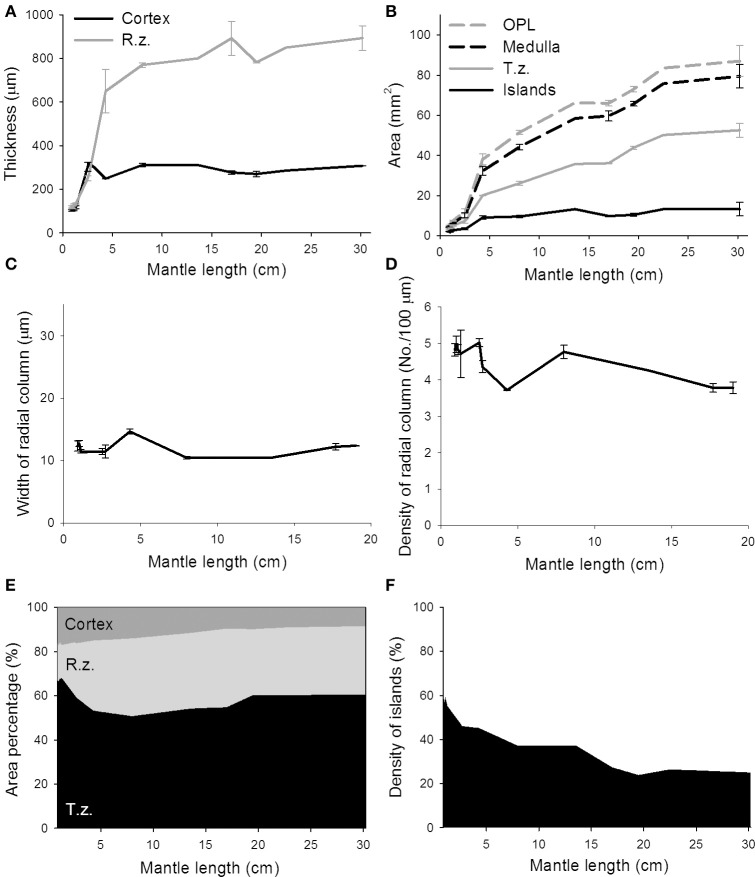
The proportion of cell islands in the medulla decreases continuously from hatching to adulthood, while the growth of the cortex and radial column zone remains stable. **(A)** The thickness of the cortex and radial column zone (R.z.) of the optic lobe increases when the mantle length of cuttlefish is less than 5 cm, but stops increasing during the sub-adult and adult stages. **(B)** The areas of the optic lobe (OPL), medulla, and tangential zone (T.z.) increase continuously throughout post-hatching development, but the area of cell islands stops increasing after the mantle length of cuttlefish exceeds 5 cm. **(C)** The width of the radial column stays relatively constant during most of post-hatching stages. **(D)** Density of the radial columns also maintains in a moderate range throughout most of post-hatching stages. **(E)** The proportion of the radial column zone increases steadily when the mantle length of cuttlefish is less than 5 cm, but decreases gradually when cuttlefish reach the adult stage. **(F)** The density of cell islands in the tangential zone of the optic lobe decreases continuously as cuttlefish develop into adulthood.

To quantify these morphological changes in the neural organization during the development of the optic lobe, the thickness of the cortex and radial column zone was measured from animals of mantle length 1.0–30.2 cm (Figure 6A). The results showed that both the cortex and radial column zone have two distinct growth phases, the first one being when the cuttlefish's mantle length is below 5 cm, and the second one being when the mantle length exceeds 5 cm. It should be noted that the relation between size and age of the animals is not strictly linear (Minton et al., 2001), thus the observed two growth phases may not correlate with the cuttlefish's age. When the cuttlefish were in the post-hatching stages, the thickness of the cortex and radial column zone increased significantly, but when the cuttlefish reached the sub-adult and adult stages, the thickness of the cortex and radial column zone remained relatively stable. A careful examination of the organization of the radial column zone revealed that both the width and the density of radial columns did not change drastically from hatching to adulthood (Figures 6C,D). When the areas of the optic lobe, medulla, tangential zone, and cell islands were compared, the results showed that the expansion of the optic lobe, medulla, and tangential zone was fast when the mantle length was below 5 cm, and continued even when cuttlefish reached sub-adult and adult stages, though the size increase in the tangential zone was relatively slower at these later stages (Figure 6B). In contrast, the expansion of the cell islands was steady during the juvenile stage, but stopped after the mantle length of cuttlefish was above 5 cm. As a result of these relative area changes, the proportion of the cortex in the optic lobe showed a continuous decrease as cuttlefish grew (Figure 6E and Table S4). However, the proportion of the radial column zone increased significantly when the mantle length was less than 5 cm, but decreased gradually when cuttlefish reached adult stage. Furthermore, the proportion of the tangential zone was increased moderately after cuttlefish reached the sub-adult and adult stages. Since the increase in cell islands was slower than that of the tangential zone, the density of cell islands in the tangential zone decreased continuously throughout the entire post-hatching development period (Figure 6F and Table S4). It should be noted that the density of cell islands decreased from 60% to about 40% when the mantle length of cuttlefish was below 5 cm, indicating that the tangential zone is transformed from cell soma dominant to neuropil dominant. In addition, the density of the cell islands continued to decrease from about 40–20% when the mantle length was above 5 cm, indicating that neural fibers among cell islands are increasing without there being significant cell proliferation during the sub-adult and adult stages.

### The development of the optic lobe is accompanied by increases of cell soma size and neural fibers

**Figure 7:**
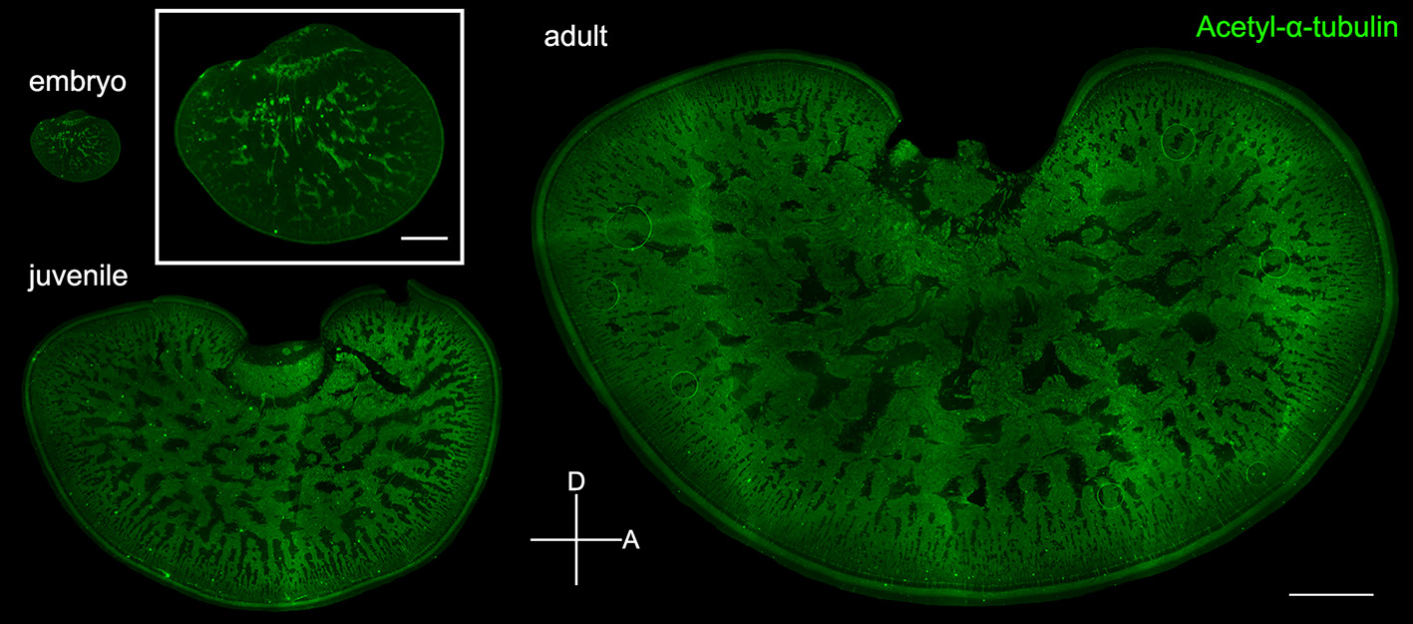
Neural fibers in the tangential zone are complementary to the cell islands in the optic lobe. Fluorescence images of acetyl-α-tubulin which labels the neural fibers in the tangential zone of embryo (stage 24), juvenile (ML = 4 cm), and adult (ML = 17.7 cm). Inset shows the enlarged image of the optic lobe in embryo. D, dorsal; A, anterior. Scale bar, 1 mm (Inset, 200 μm).

To verify the observation that the expansion of the neuropil area is indeed a result of the increase of neural processes in the tangential zone, acetyl-α-tubulin which labels neural fibers was used to visualize the development of the optic lobe in embryonic, juvenile, and adult cuttlefish (Figure 7). Complementary to the images in Figures 3, 5 which showed the distribution of the cell somata, immunostaining images of acetyl-α-tubulin revealed neural processes in the optic lobe. It is apparent that the neural fibers increased continuously in the tangential zone throughout different developmental stages. To distinguish the origin of the increased neural fibers in the tangential zone during development, the neural fibers in the input region (the cortex and radial column zone), the tangential zone, and the output region (the optic tract region) of the optic lobe were characterized separately at three different developmental stages. It was found that the proportion of neural fibers of the tangential zone remained stable throughout developmental stages (Figure S1 and Table S5). This suggests that the increase of neural fibers in the tangential zone as cuttlefish growing is equally contributed by the fibers from the input region, tangential zone, and output region of the optic lobe.

**Figure 8:**
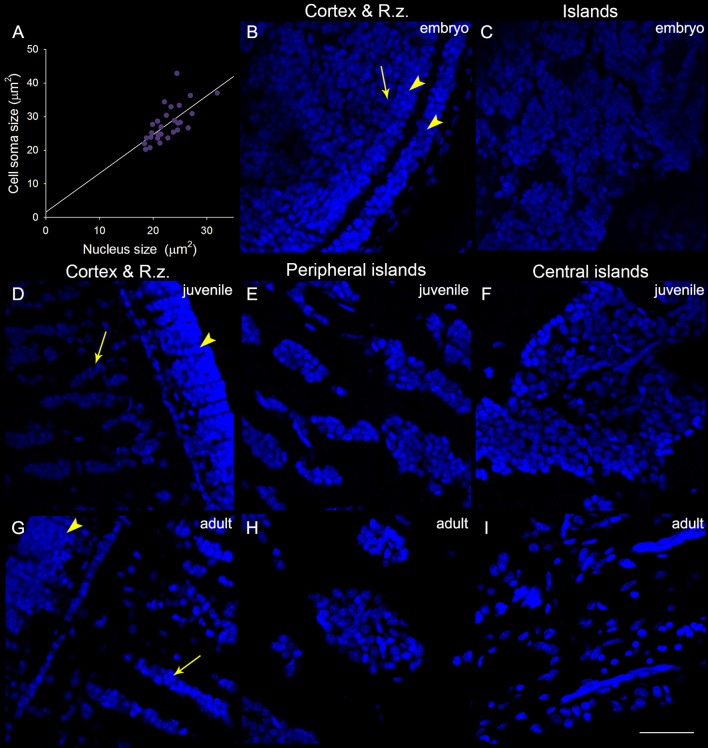
The organization and cell size within the optic lobe change significantly from the embryonic stage to the adulthood. **(A)** A positive correlation (*R*^2^ = 0.454, *p* < 0.001) between the nucleus size (estimated from the DAPI staining images) and the cell soma size (estimated from the acetyl-α-tubulin staining images and confirmed with the DIC images) obtained from the measurements (30 nuclei) of the cell islands in a juvenile cuttlefish (ML = 4 cm). This relationship allows us to use the nucleus size as a proxy to estimate the cell soma size in the cell islands. **(B)** The DAPI staining image of the cortex and radial column zone (R.z.) at the embryonic stage 24. **(C)** Nuclei (DAPI staining) in the tangential zone are not yet separable into the cell islands at the embryonic stage 24. **(D–F)** The DAPI staining images of the input region (the cortex and radial column zone) as well as cell islands in the peripheral and central regions of the optic lobe from a juvenile cuttlefish (ML = 4 cm), respectively. **(G–I)** The DAPI staining images of the input region as well as cell islands in the peripheral and central regions of the optic lobe from an adult cuttlefish (ML = 17.7 cm), respectively. Scale bar, 50 μm; Arrowhead, the granular layer of the cortex; Arrow, the stacked nuclei in the radial column zone.

**Figure 9:**
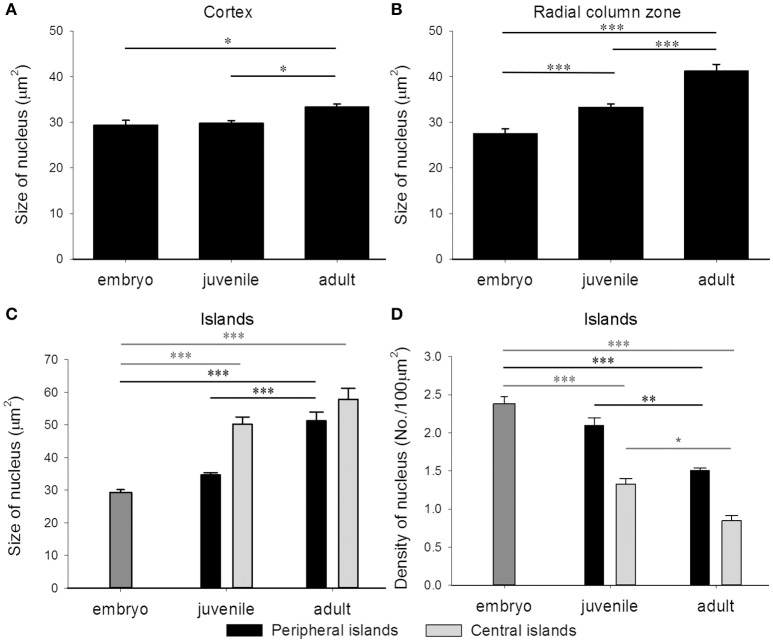
The nucleus size in the optic lobe increases while the nucleus density in the cell islands decreases during development. **(A)** The average nucleus size in the cortex of the optic lobe increases significantly from juvenile to adult cuttlefish. **(B)** The average nucleus size in the radial column zone of the optic lobe increases significantly throughout all developmental stages of cuttlefish. **(C)** Due to the volume difference of the cell islands at various regions of the tangential zone in juvenile and adult cuttlefish, the peripheral and central regions were analyzed separately. The average nucleus size in the cell islands of the peripheral region increases significantly from juvenile to adult cuttlefish. In contrast, the average nucleus size in the cell islands of the central region increases significantly from embryo to juvenile cuttlefish. **(D)** The average nucleus density in the cell islands of the peripheral region decreases significantly from juvenile to adult cuttlefish. In contrast, the average nucleus density in the cell islands of the central region decreases significantly throughout all three developmental stages. ^***^p < 0.001; ^**^p < 0.01; ^*^p < 0.05.

In addition to the increase of neural fibers during development, the cell soma size in different areas of the optic lobe was also expanded. It is apparent that the cell soma size and cell density in the cell islands appeared smaller and packed, respectively, when cuttlefish were in earlier developmental stages (Figure 8). This observation indicates that the organization and cell size within the cell islands of the optic lobe change continuously from the embryonic stage to the adulthood. To quantify the morphological and organizational changes down to the cellular level in the optic lobe during development, the nucleus size (a proxy to estimate the cell soma size) and the nucleus density in the cell islands were estimated. The nucleus size in the cortex increased only from juvenile to adult cuttlefish (Figure 9A), while that of the radial column zone increased significantly from embryonic stage to adult cuttlefish (Figure 9B). In addition, it is evident that the average nucleus size in the cell islands of the peripheral region increased significantly from juvenile to adult cuttlefish (Figure 9C). In contrast, the average nucleus size in the cell islands of the central region increased significantly from embryo to juvenile cuttlefish. Similarly, the average nucleus density in the cell islands of the peripheral region decreased significantly from juvenile to adult cuttlefish (Figure 9D). In contrast, the average nucleus density in the cell islands of the central region decreased significantly throughout all three developmental stages. These findings suggest that the entire optic lobe, especially the cell islands, have gone through a significant change during development and this reorganization in the optic lobe may be important for behavioral changes throughout the life.

### Lateralization of the optic lobes is evident in the cortex and radial column zone during the embryonic and juvenile stages

**Figure 10:**
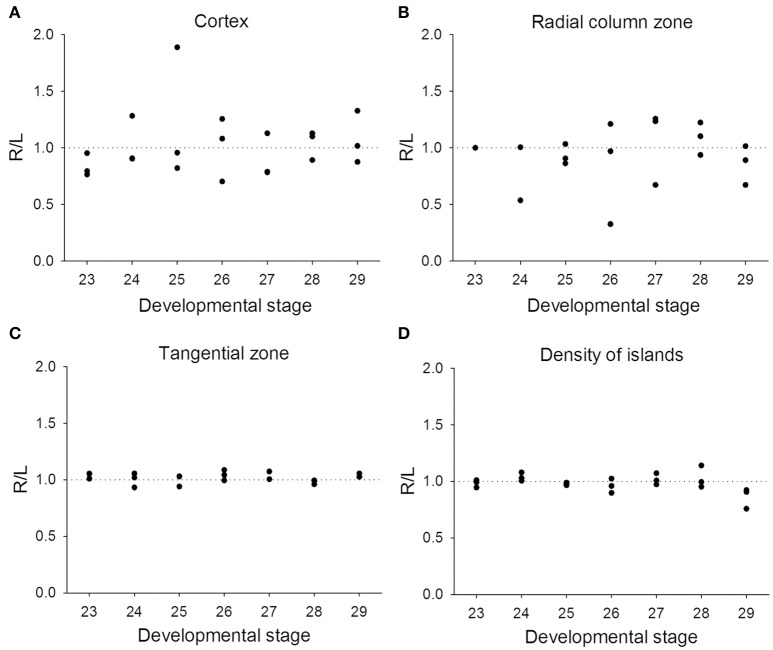
Lateralization of the optic lobe is more pronounced in the cortex and radial column zone during the embryonic stages. The lateralization index is defined as Right/Left. **(A,B)** The relative areas of the cortex and radial column zone show a large variation in lateralization index throughout all late embryonic stages. **(C,D)** The relative area of the tangential zone and the density of cell islands are mostly symmetric across the various embryonic stages.

**Figure 11:**
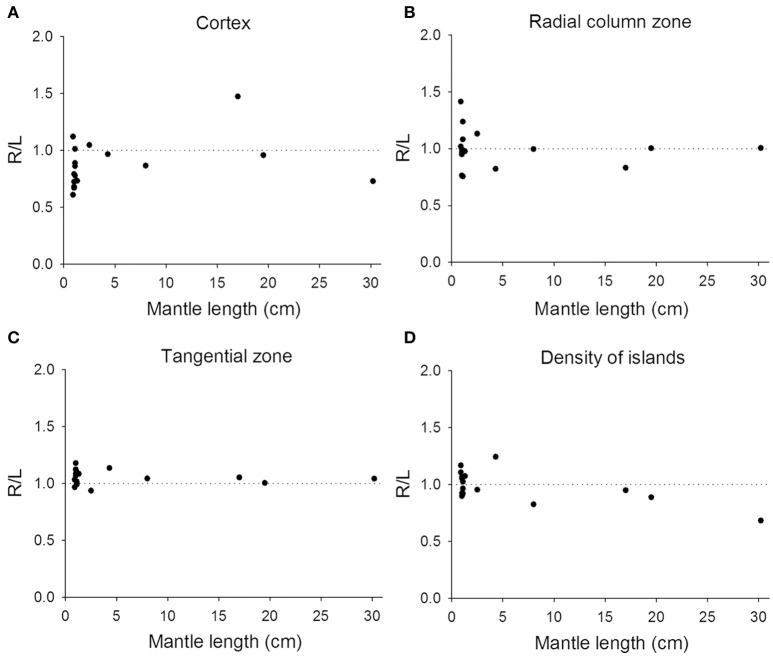
Lateralization of the optic lobe is more apparent in juvenile cuttlefish. **(A,B)** The relative areas of the cortex and radial column zone show a large variation in the lateralization index when the mantle length of cuttlefish is less than 5 cm. **(C,D)** The relative area of the tangential zone and the density of cell islands have a reduced lateralization index variation compared to the cortex and radial column zone.

Previous studies on visual lateralization in the common European cuttlefish *S. officinalis* found that the sizes of the left and right optic lobes were not identical and this cerebral asymmetry creates a bias with respect to their side-turning behavior (Jozet-Alves et al., 2012a,b). In the present study, although the developmental pace of the left and right optic lobes in pharaoh cuttlefish *S. pharaonis* was similar at various life stages (Table S2), we found that the internal structures of the optic lobe in *S. pharaonis* showed a prominent variability in left/right asymmetry when the two sides of the brain were compared throughout the embryonic and juvenile stages (Figures 10, 11). Specifically, the relative areas of the cortex and radial column zone showed a large variation with respect to the lateralization index throughout all late embryonic stages (Figures 10A,B) and when the mantle length of cuttlefish was less than 5 cm (Figures 11A,B). Interestingly, the internal structures of the optic lobe on the two sides of the brain became more symmetrical during the sub-adult and adult stages. Note that the sample size for the ML > 5 cm was small, thus the data should be treated cautiously. In contrast, the relative area of the tangential zone and the density of the cell islands were largely symmetrical during the late embryonic stages (Figures 10C,D) and throughout all post-hatching life (Figures 11C,D). These findings regarding the optic lobe indicate that the visual processing areas (the cortex and radial column zone) are more prone to lateralization than the visuomotor control area (the tangential zone) during the development of the cuttlefish.

## Discussion

### Ontogenetic development of the optic lobe

The present study reveals that the maturation of the various different regions in the optic lobe of cuttlefish is a non-uniform process. Specifically, morphological changes are most significant around embryonic stage 24, hatching, and at the time when the mantle length reaches 5 cm (Figures 4, 6). In other words, the basic neural organization of the cuttlefish optic lobe is established when the animals are still young, but neural fibers among cell islands increase continuously from juvenile to adulthood. This type of brain maturation pattern is common in other animals. For example, mice complete neuronal differentiation and migration within the cerebral cortex a few days before birth, while neural connections among the various different cortical areas continue to form well beyond birth (Johnson et al., 2002). It has also been reported that most neurons in the ventral nerve cord of adult fruit flies are created during the larval stage, but the neural connections are continuously increased during later developmental stages (Truman and Bate, 1988).

Cuttlefish are a semelparous species, which means that eggs and juveniles develop without parental care. As a consequence, eggs are very vulnerable, and hatchlings need to cope on their own to find food and avoid predators. At embryonic stage 24, the layered structure of the optic lobe becomes evident with the first appearance of the radial column zone (Figure 3). This time point corresponds roughly to the observation that the visual system is functional from stage 25 of the cuttlefish *S. officinalis* (Romagny et al., 2012). More importantly, it is known that embryonic visual experience has a significant impact on the development of post-hatching behavior (Darmaillacq et al., 2006, 2008; Guibe and Dickel, 2011). Thus, early development of the visual processing area within the optic lobe, including the cortex and radial column zone, is crucial to allowing cuttlefish to detect visual stimuli and adapt to different visual environments very early in life. In addition, the finding that the radial columns decrease the width and increase the density throughout embryonic development (Figures 4C,D) suggests that the spatial resolution of the visual information processing is also continuously increased before hatching. Future studies of examining the effect of visual deprivation on the development of optic lobes and visual behaviors of hatchlings will provide further evidence of neural plasticity at this critical stage.

From hatching to a mantle length of 5 cm is another fast growing period that involves a significant increase in the size of the optic lobe (Figure 5). However, much of this size increase in the optic lobe is attributable to an expansion of the medulla rather than of the cortex. Although previous developmental studies of the squid's brain have shown that the neuropil appear earlier in the tangential zone than in the radial column zone (Shigeno et al., 2001b,c; Yamamoto et al., 2003), the area of radial column zone actually grows faster than that of the tangential zone before the mantle length reaches 5 cm (Figure 6). Despite the slower expansion rate of the tangential zone, this area is gradually transformed from cell soma dominant to neuropil dominant. These results suggest that neural fibers among cell islands are increasing without significant cell proliferation. It has been well documented that post-hatching visual experience is important to shape the visual behavior of juvenile cuttlefish (Dickel et al., 2000; Poirier et al., 2005; Lee et al., 2010, 2012). The finding that the radial column zone and neural fibers among cell islands are disproportionally increased during this post-hatching period further supports the hypothesis that visual perception and visuomotor control of body patterning are crucial to juvenile cuttlefish as part of their camouflage and other defensive behaviors (Hanlon and Messenger, 1988).

After the mantle length reaches 5 cm and beyond, the optic lobe of cuttlefish continues to grow, but much of the size increase is a result of tangential zone growth rather than radial column zone growth (Figure 6). Furthermore, the density of the cell islands continuously decreases, suggesting that neural fibers among cell islands are increasing continuously without significant cell proliferation during the sub-adult and adult stages. It is known that the function of body patterning in cuttlefish continuously changes from hatching to adulthood; specifically there is a change from primarily being used for concealment as a defensive behavior to being mainly used for visual communication during reproductive behavior (Hanlon and Messenger, 1996). The observation that the tangential zone and the neural fibers among the cell islands are increased during the sub-adult and adult stages further supports the hypothesis that visuomotor control of dynamic body patterning depends on neural processing among the cell islands in the medulla of the optic lobe. These results are similar to studies on birds in which it has been found that the brain size is highly correlated with the development of novel foraging techniques (Overington et al., 2009). Furthermore, it has been shown that the volume of the song-related nuclei and the size of the associated neural tissues in songbirds are correlated with their song length and repertoire size (Garamszegi and Eens, 2004). These examples support a strong correlation between neural reorganization and behavioral modification throughout the life of animals.

In a close examination of cell morphology and fibers distribution in the optic lobe (Figure 7), we found that the proportion of neural fibers of the tangential zone remains stable throughout developmental stages (Figure S1). This suggests that the origin of the increased neural fibers observed in the tangential zone during development (Figures 4, 6) is equally contributed by the growth of neural processes from the input and output regions as well as within the tangential zone itself. It also supports that information transfer in and out of the tangential zone is increased proportionally with the information processing within the tangential zone during cuttlefish development. Surprisingly, by examining the nucleus size in different areas of the optic lobe and the density of the cell islands at different developmental stages, we found that the organization and cell size within the optic lobe change significantly from the embryonic stage to adulthood (Figures 8, 9). This finding suggests that specific areas in the optic lobe, especially the cell islands, are continuously reorganized to account for behavioral changes throughout life. It has been reported that the robust nucleus of the archistriatum (RA), an anatomically discrete brain region that is known to be involved with song production in birds, increase greatly in volume during a restricted period of song development in male zebra finches, and the growth of the RA is due to an increase in the cell soma size and a decrease in the cell density (Bottjer et al., 1986). This result suggests that the cells in the RA are undergoing fundamental maturational changes as the song behavior is beginning to acquire its adult form. This observation in the RA of zebra finches is parallel with our finding in the optic lobe of cuttlefish. The increase in the cell size may indicate that the metabolic activity of these neurons is increasing, whereas the decrease in the cell density may indicate that the dendritic arbor of these neurons is increasing, and/or that neural fibers from other regions are growing into this area. Further studies are needed to elucidate the underlying mechanism of morphological changes in the optic lobe during cuttlefish development.

### Neural basis of visual lateralization

In the present study, we confirmed that lateralization of the optic lobe may be a general feature of cuttlefish and that it is age dependent. Our results are consistent with the previous study showing that there is individual variation in the magnitude of the optic lobe asymmetry among the common European cuttlefish *S. officinalis* (Jozet-Alves et al., 2012a), and we have also shown that the lateralization indices vary greatly in both the cortex and radial column zone during the embryonic and juvenile stages of the pharaoh cuttlefish *S. pharaonis*. In contrast to the above, the variation was much less in the tangential zone and in the density of the cell islands (Figures 10, 11). In the aforementioned study, these authors found that these anatomical brain asymmetries were correlated with behavioral asymmetries, that is the larger the right optic lobe, the stronger the bias toward turning leftwards (Jozet-Alves et al., 2012a). In a separate study, it was also suggested that this left-turning bias observed in juvenile cuttlefish was the result of an eye use preference (Jozet-Alves et al., 2012b). Although, we did not carry out behavioral experiments to examine the correlation between anatomical asymmetry and turning bias, the fact that the cortex and radial column zone (the visual processing areas) are more prone to lateralization than the tangential zone (the visuomotor control area) in the optic lobe of developing cuttlefish supports the idea that eye use preference brings about the visual experience-dependent enhancement of side-turning preference. This is akin to neural plasticity observed in primate visual cortex and frog tectum (Hubel and Wiesel, 1977; Constantine-Paton and Law, 1978).

### Neural organization of the optic lobe

Earlier morphological studies of the optic lobes in octopus and squid (Young, 1962, 1974) suggested that the columnar organization of the radial column zone is likely to retain retinotopic information and thus it functions as a visual feature processing center. This neural organization pattern is similar to the visual cortex of the mammalian brain (Hubel and Wiesel, 1977), and the lamina and outer medulla of the insect's optic lobe (Strausfeld, 1970), which suggests a convergent evolution of these animal's brain organizations. Despite this similarity, there are some obvious differences in their neural systems (Breidbach and Kutsch, 1995). For example, the axons from the retina of cephalopods and insects do not form a bundle before projecting to the optic lobe, and the dorsoventral chiasma located behind the eye of cephalopods reverses the optically inverted image back in the optic lobe (Young, 1974). This latter feature ensures that the cortex and radial column zone in cephalopods retain upright retinotopic information, unlike the one in the visual cortex of mammalian brains. Furthermore, the optic nerves are mostly projected to the ipsilateral side of the optic lobe in cephalopods and insects, while they are largely projected to the contralateral side of the brain in vertebrates (Nixon and Young, 2003). These observations suggest that the visual system of cephalopods and insects are more alike when compared with vertebrates, despite the convergent evolution of the optics of the eyes in cephalopods and vertebrates (Packard, 1972).

In the tangential zone during the present study the so called “cell islands” (Young, 1962, 1974) were found to be contiguous in the MRI data, forming a tree-like structure (Figure 2A). Since the internal organization of the optic lobe is quite conserved in most species of cephalopods (Nixon and Young, 2003), this 3D structure of the optic lobe from a sub-adult cuttlefish suggests that such an organization of neurons within the tangential zone could be a general feature in all optic lobes of cephalopods (Boycott, 1961; Young, 1962, 1974). By electrically stimulating the medulla of the optic lobe, previous studies have shown that various body patterns can be evoked unilaterally or bilaterally in cuttlefish (Boycott, 1961). Further studies have shown that the medulla is responsible for producing various types of locomotive behavior (Chichery and Chanelet, 1976, 1978). Our recent study also supports that various body pattern components can be selectively activated together when the medulla of the optic lobe from the oval squid *Sepioteuthis lessoniana* was stimulated electrically (Liu and Chiao, 2017). These findings suggest that the medulla, specifically the tangential zone, is the motor command center for locomotion and dynamic body patterning in cephalopods (Messenger, 2001). The motor signals generated by the medulla are the major input for the basal lobe, which is also an important motor controlling center in cephalopods and have been suggested to be functionally similar to the basal ganglia in vertebrates (Gleadall, 1990). Collectively, the functions of the tangential zone of the optic lobe and the basal lobe in cephalopods seem to be similar to that of the basal ganglia in vertebrates, where the primary function is to receive the sensory signals from various cortical layer, and generate motor signals to downstream brain structures for controlling and regulating the activities of the motor and premotor cortical areas during voluntary movements (Alexander et al., 1986; Reiner et al., 1998). However, the neural organizations of these two motor command centers are significantly different. In the optic lobe of cephalopods, neurons are clustered together and formed a contiguous tree-like structure, whereas neural organization in the striatum of basal ganglia have no apparent regional differences despite of the perfect topography and neurotransmitter-related neuronal distribution in different regions or nuclei (Squire et al., 2003). Nevertheless, both brain structures receive information from the sensory areas and select actions by sending control signals to the motor areas. Thus, the optic lobe with the downstream basal lobe in cephalopods and the basal ganglia in vertebrates are both crucial to the control of the motor movements required for specific behaviors. Alternatively, it has been suggested that the optic tectum of vertebrates, particularly fish and amphibians, has many output neurons to the midbrain territories that regulate motor programs (reaction, orientation, attack, and escape, etc.), thus the function and projection might be similar between the optic lobe in cephalopods and the optic tectum in fish and amphibians (Butler and Hodos, 2005). Finally, it has been suggested that the arthropod central complex and vertebrate basal ganglia circuitries that underlie the selection and maintenance of behavioral actions are evolutionarily conserved (Strausfeld and Hirth, 2013; Fiore et al., 2015). Thus it is possible that the optic lobe of cephalopods shares deep homology with the central complex of arthropods and the basal ganglia of vertebrates.

## Ethics statement

This study was exempt from one or more of the above requirements, because cephalopods are invertebrates and do not need to be approved by the institutional animal care and use committee in Taiwan.

## Author contributions

YL conceived, designed, carried out the work, and drafted the manuscript. TL helped plan experiments and interpreted data. CS collected MRI data. CC helped plan experiments, interpreted data, and revised the manuscript.

### Conflict of interest statement

The authors declare that the research was conducted in the absence of any commercial or financial relationships that could be construed as a potential conflict of interest.

## Supplementary material

Figure S1 Proportions of neural fibers of the input region (the cortex and radial column zone), the tangential zone (T.z.), and the output region (the optic tract region) of the optic lobe have no significant difference among different developmental stages.

Table S1 The optic lobe samples and their nuclear staining methods.

Table S2 All measurements from the left and right optic lobes are monotonically related.

Table S3 Standard errors not shown in Figures 4E,F.

Table S4 Standard errors not shown in Figures 6E,F.

Table S5 Standard errors not shown in Figure S1.

Movie S1 A pair of stereo images enhanced and reconstructed from the MRI scan showing the 3D structure of cell islands (light areas) in the medulla of the left optic lobe of a cuttlefish (ML = 16 cm). This MRI scan reveals for the first time that the so called “cell islands” present in the medulla (Young, 1962, 1974) are contiguous and have a tree-like structure.

## References

[B1] Alexander G. E. Delong M. R. Strick P. L. (1986). Parallel organization of functionally segregated circuits linking basal ganglia and cortex. Annu. Rev. Neurosci. 9, 357–381. 10.1146/annurev.ne.09.030186.002041 3085570

[B2] Bottjer S. W. Miesner E. A. Arnold A. P. (1986). Changes in neuronal number, density and size account for increases in volume of song-control nuclei during song development in zebra finches. Neurosci. Lett. 67, 263–268. 10.1016/0304-3940(86)90319-8 3737014

[B3] Boycott B. B. (1961). Functional organization of brain of cuttlefish Sepia officinalis. Proc. R. Soc. Ser. B Bio. 153, 503–534. 10.1098/rspb.1961.0015

[B4] Boyle P. R. (1983). Cephalopod Life Cycles. London; New York, NY: Academic Press.

[B5] Breidbach O. Kutsch W. (1995). The Nervous Systems of Invertebrates: An Evolutionary and Comparative Approach. Birkhäuser Basel.

[B6] Butler A. B. Hodos W. (2005). Comparative Vertebrate Neuroanatomy: Evolution and Adaptation. Hoboken, NJ: Wiley-Interscience.

[B7] Cajal S. R. (1917). Contribucion al conocimiento de la retina y centros opticos de los cefalopodos. Trab. Lab. Invest. Biol. Univ. Madr. 15, 1–82.

[B8] Chichery R. Chanelet J. (1976). Motor and behavioral-responses obtained by stimulation with chronic electrodes of optic lobe of Sepia officinalis. Brain Res. 105, 525–532. 10.1016/0006-8993(76)90598-9 1260459

[B9] Chichery R. Chanelet J. (1978). Motor-responses obtained by stimulation of peduncle lobe of Sepia officinalis in chronic experiments. Brain Res. 150, 188–193. 10.1016/0006-8993(78)90664-9 667619

[B10] Constantine-Paton M. Law M. I. (1978). Eye-specific termination bands in tecta of three-eyed frogs. Science 202, 639–641. 10.1126/science.309179 309179

[B11] Darmaillacq A. S. Chichery R. Shashar N. Dickel L. (2006). Early familiarization overrides innate prey preference in newly hatched Sepia officinalis cuttlefish. Anim. Behav. 71, 511–514. 10.1016/j.anbehav.2005.04.019

[B12] Darmaillacq A. S. Lesimple C. Dickel L. (2008). Embryonic visual learning in the cuttlefish, *Sepia officinalis*. Anim. Behav. 76, 131–134. 10.1016/j.anbehav.2008.02.006

[B13] Dickel L. Boal J. G. Budelmann B. U. (2000). The effect of early experience on learning and memory in cuttlefish. Dev. Psychobiol. 36, 101–110. 10.1002/(SICI)1098-2302(200003)36:2<101::AID-DEV2>3.0.CO;2-L 10689281

[B14] Fiore V. G. Dolan R. J. Strausfeld N. J. Hirth F. (2015). Evolutionarily conserved mechanisms for the selection and maintenance of behavioural activity. Philos. T. R. Soc. B 370:20150053. 10.1098/rstb.2015.0053 26554043 PMC4650127

[B15] Garamszegi L. Z. Eens M. (2004). Brain space for a learned task: strong intraspecific evidence for neural correlates of singing behavior in songbirds. Brain Res. Rev. 44, 187–193. 10.1016/j.brainresrev.2003.12.001 15003393

[B16] Gleadall I. G. (1990). Higher motor function in the brain of Octopus: the anterior basal lobe and its analogies with the vertebrate basal ganglia. Ann. Appl. Inf. Sci. 16, 1–30.

[B17] Guibe M. Dickel L. (2011). Embryonic visual experience influences post-hatching shelter preference in cuttlefish. Vie Milieu 61, 243–246.

[B18] Hanlon R. T. Messenger J. B. (1988). Adaptive coloration in young cuttlefish (*Sepia officinalis* L) - the morphology and development of body patterns and their relation to behavior. Philos. T. R. Soc. B 320, 437–487. 10.1098/rstb.1988.0087

[B19] Hanlon R. T. Messenger J. B. (1996). Cephalopod Behaviour. Cambridge; New York, NY: University of Cambridge.

[B20] Hubel D. H. Wiesel T. N. (1977). Functional architecture of macaque monkey visual-cortex. Proc. R. Soc. Ser. B Bio. 198, 1–59. 10.1098/rspb.1977.0085 20635

[B21] Johnson M. H. Munakata Y. Gilmore R. O. (2002). Brain Development and Cognition: A Reader. Oxford; Malden, MA: Blackwell Publishers.

[B22] Jozet-Alves C. Romagny S. Bellanger C. Dickel L. (2012a). Cerebral correlates of visual lateralization in Sepia. Behav. Brain Res. 234, 20–25. 10.1016/j.bbr.2012.05.042 22677275

[B23] Jozet-Alves C. Viblanc V. A. Romagny S. Dacher M. Healy S. D. Dickel L. (2012b). Visual lateralization is task and age dependent in cuttlefish, Sepia officinalis. Anim. Behav. 83, 1313–1318. 10.1016/j.anbehav.2012.02.023

[B24] Kerbl A. Handschuh S. Nodl M. T. Metscher B. Walzl M. Wanninger A. (2013). Micro-CT in cephalopod research: investigating the internal anatomy of a sepiolid squid using a non-destructive technique with special focus on the ganglionic system. J. Exp. Mar. Biol. Ecol. 447, 140–148. 10.1016/j.jembe.2013.02.022

[B25] Klagges B. R. E. Heimbeck G. Godenschwege T. A. Hofbauer A. Pflugfelder G. O. Reifegerste R.. (1996). Invertebrate synapsins: a single gene codes for several isoforms in Drosophila. J. Neurosci. 16, 3154–3165. 8627354 10.1523/JNEUROSCI.16-10-03154.1996 PMC6579133

[B26] Kobayashi S. Takayama C. Ikeda Y. (2013). Ontogeny of the brain in oval squid *Sepioteuthis lessoniana* (Cephalopoda: Loliginidae) during the post-hatching phase. J. Mar. Biol. Assoc. U.K. 93, 1663–1671. 10.1017/S0025315413000088

[B27] Lee M. F. Lin C. Y. Chiao C. C. Lu C. C. (2016). Reproductive behavior and embryonic development of the pharaoh cuttlefish, Sepia pharaonis (Cephalopoda: Sepiidae). Zool. Stud. 55:41 10.6620/ZS.2016.55-41 PMC6511901 31966186

[B28] Lee Y. H. Yan H. Y. Chiao C. C. (2010). Visual contrast modulates maturation of camouflage body patterning in cuttlefish (*Sepia pharaonis*). J. Comp. Psychol. 124, 261–270. 10.1037/a0019461 20695657

[B29] Lee Y. H. Yan H. Y. Chiao C. C. (2012). Effects of early visual experience on the background preference in juvenile cuttlefish Sepia pharaonis. Biol. Lett. 8, 740–743. 10.1098/rsbl.2012.0398 22791707 PMC3440992

[B30] Lemaire J. (1970). Table de développement embryonnaire de *Sepia officinalis* L.(Mollusque Céphalopode). Bull. Soc. Zool. Fr. 95, 773–782.

[B31] Liu T. H. Chiao C. C. (2017). Mosaic organization of body pattern control in the optic lobe of squids. J. Neurosci. 37, 768–780. 10.1523/JNEUROSCI.0768-16.2016 28123014 PMC6597019

[B32] Marquis V. F. (1989). Die embryonalentwicklung des nervensystems von *Octopus vulgaris* lam. (Cephalopoda, Octopoda), eine histologische analyse. Verh. Naturforsch. Ges. Basel. 99, 23–75.

[B33] Meister G. (1972). Organogenese von Loligo vulgaris LAM: Mollusca, Cephalopoda, Teuthoidea, Myopsida, Loliginidae. Zool. Jb. Anat. 89, 247–300.

[B34] Messenger J. B. (2001). Cephalopod chromatophores: neurobiology and natural history. Biol. Rev. Camb. Philos. Soc. 76, 473–528. 10.1017/S1464793101005772 11762491

[B35] Minton J. W. Walsh L. S. Lee P. G. Forsythe J. W. (2001). First multi-generation culture of the tropical cuttlefish *Sepia pharaonis* Ehrenberg, 1831. Aquacult. Int. 9, 379–392. 10.1023/A:1020535609516

[B36] Mooney T. A. Lee W. J. Hanlon R. T. (2010). Long-duration anesthetization of squid (*Doryteuthis pealeii*). Mar. Freshw. Behav. Phy. 43, 297–303. 10.1080/10236244.2010.504334

[B37] Nixon M. Young J. Z. (2003). The Brains and Lives of Cephalopods. Oxford; New York, NY: Oxford University Press.

[B38] O'Dor R. K. Webber D. M. (1986). The constraints on cephalopods - why squid arent fish. Can. J. Zool. 64, 1591–1605. 10.1139/z86-241

[B39] Overington S. E. Morand-Ferron J. Boogert N. J. Lefebvre L. (2009). Technical innovations drive the relationship between innovativeness and residual brain size in birds. Anim. Behav. 78, 1001–1010. 10.1016/j.anbehav.2009.06.033

[B40] Packard A. (1972). Cephalopods and fish: the limits of convergence. Biol. Rev. 47, 241–307. 10.1111/j.1469-185X.1972.tb00975.x

[B41] Poirier R. Chichery R. Dickel L. (2005). Early experience and postembryonic maturation of body patterns in cuttlefish (*Sepia officinalis*). J. Comp. Psychol. 119, 230–237. 10.1037/0735-7036.119.2.230 15982166

[B42] Reiner A. Medina L. Veenman C. L. (1998). Structural and functional evolution of the basal ganglia in vertebrates. Brain Res. Rev. 28, 235–285. 10.1016/S0165-0173(98)00016-2 9858740

[B43] Romagny S. Darmaillacq A. S. Guibe M. Bellanger C. Dickel L. (2012). Feel, smell and see in an egg: emergence of perception and learning in an immature invertebrate, the cuttlefish embryo. J. Exp. Biol. 215, 4125–4130. 10.1242/jeb.078295 23136152

[B44] Shigeno S. Kidokoro H. Tsuchiya K. Segawa S. Yamamoto M. (2001a). Development of the brain in the oegopsid squid, Todarodes pacificus: an atlas from hatchling to juvenile. Zool. Sci. 18, 1081–1096. 10.2108/zsj.18.1081

[B45] Shigeno S. Kidokoro H. Tsuchiya K. Segawa S. Yamamoto M. (2001b). Development of the brain in the oegopsid squid, Todarodes pacificus: an atlas up to the hatching stage. Zool. Sci. 18, 527–541. 10.2108/zsj.18.527

[B46] Shigeno S. Tsuchiya K. Segawa S. (2001c). Embryonic and paralarval development of the central nervous system of the loliginid squid *Sepioteuthis lessoniana*. J. Comp. Neurol. 437, 449–475. 10.1002/cne.1295 11503146

[B47] Shigeno S. Yamamoto M. (2002). Organization of the nervous system in the pygmy cuttlefish, *Idiosepius paradoxus* Ortmann (Idiosepiidae, Cephalopoda). J. Morphol. 254, 65–80. 10.1002/jmor.10020 12219344

[B48] Squire L. R. Bloom F. E. McConnell S. K. Roberts J. L. Spitzer N. C. Zigmond M. J. (2003). Fundamental Neuroscience. Amsterdam; San Diego, CA; London: Academic Press.

[B49] Strausfeld N. J. (1970). The optic lobes of Diptera. Philos. T. R. Soc. B. 258, 135–223. 10.1098/rstb.1970.0033 22408826

[B50] Strausfeld N. J. Hirth F. (2013). Deep homology of arthropod central complex and vertebrate basal ganglia. Science 340, 157–161. 10.1126/science.1231828 23580521

[B51] Truman J. W. Bate M. (1988). Spatial and temporal patterns of neurogenesis in the central nervous-system of *Drosophila melanogaster*. Dev. Biol. 125, 145–157. 10.1016/0012-1606(88)90067-X 3119399

[B52] Wollesen T. Loesel R. Wanninger A. (2009). Pygmy squids and giant brains: mapping the complex cephalopod CNS by phalloidin staining of vibratome sections and whole-mount preparations. J. Neurosci. Meth. 179, 63–67. 10.1016/j.jneumeth.2009.01.021 19428513

[B53] Yamamoto M. Shimazaki Y. Shigeno S. (2003). Atlas of the embryonic brain in the pygmy squid, *Idiosepius paradoxus*. Zool. Sci. 20, 163–179. 10.2108/zsj.20.163 12655180

[B54] Young J. Z. (1962). Optic lobes of *Octopus vulgaris*. Philos. T. R. Soc. B. 245, 19–58. 10.1098/rstb.1962.0005

[B55] Young J. Z. (1974). The central nervous system of Loligo. I. The optic lobe. Philos. T. R. Soc. B 267, 263–302. 10.1098/rstb.1974.0002 4132206

