# Supplementary figures and images for: Neural Organization of the Optic Lobe Changes Steadily from Late Embryonic Stage to Adulthood in Cuttlefish Sepia pharaonis
Source: Front Physiol. 2017 Jul 27;8:538. doi: 10.3389/fphys.2017.00538 (PMC5529416; doi:10.3389/fphys.2017.00538)

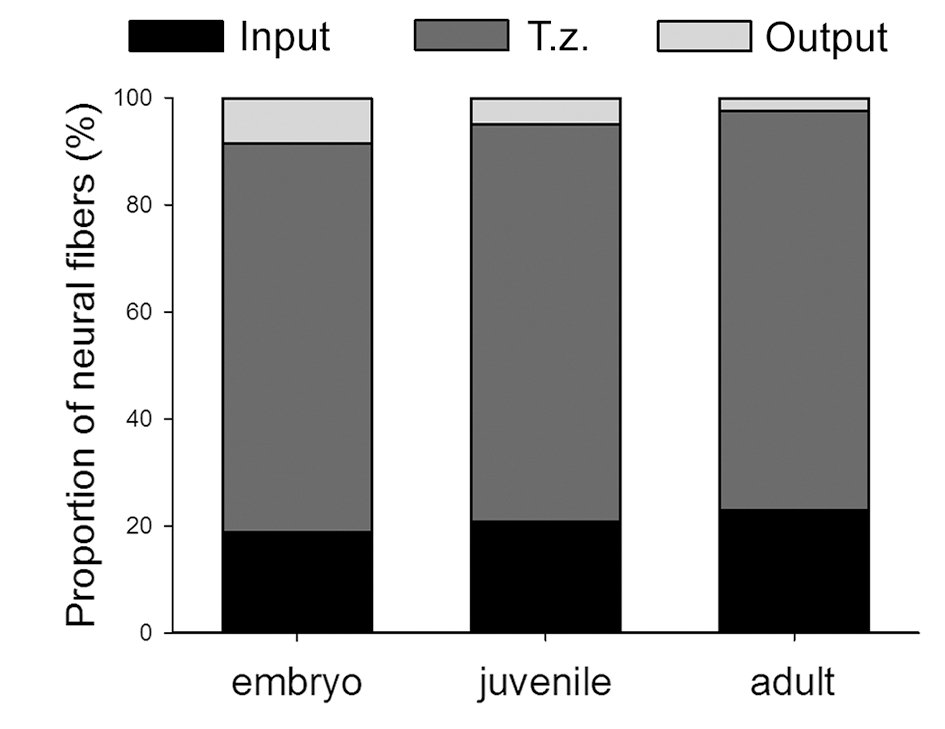

Supplement: Figure S1 — Proportions of neural fibers of the input region (the cortex and radial column zone), the tangential zone (T.z.), and the output region (the optic tract region) of the optic lobe have no significant difference among different developmental stages. [file FigureS1.TIF]
