# Supplementary material for: Neural Organization of the Optic Lobe Changes Steadily from Late Embryonic Stage to Adulthood in Cuttlefish Sepia pharaonis
Source: Front Physiol. 2017 Jul 27;8:538. doi: 10.3389/fphys.2017.00538 (PMC5529416; doi:10.3389/fphys.2017.00538)
Supplement: Table S1 — The optic lobe samples and their nuclear staining methods. [file Table1.pdf]

Table S1. The optic lobe samples and their nuclear staining methods.

Age	ML (cm)	Stage	N*	Staining
st. 22	-	embryo	2	DAPI
st. 23	-	embryo	6	DAPI
st. 24	-	embryo	6	DAPI
st. 25	-	embryo	6	DAPI
st. 26	-	embryo	6	DAPI
st. 27	-	embryo	6	DAPI
st. 28	-	embryo	6	DAPI
st. 29	-	embryo	6	DAPI
hatchling	-	hatchling	3	DAPI
-	0.9	juvenile	4	DAPI
-	1.0	juvenile	2	DAPI
-	1.0	juvenile	6	DAPI
-	1.1	juvenile	6	DAPI
-	1.1	juvenile	2	DAPI
-	1.3	juvenile	4	DAPI
-	2.5	juvenile	3	DAPI
-	2.7	juvenile	1	DAPI
-	4.3	juvenile	2	PI
-	8.0	sub-adult	2	PI
-	13.6	sub-adult	1	PI
-	17.0	sub-adult	2	PI
-	19.5	adult	2	PI
-	22.5	adult	1	PI
-	30.2	adult	2	PI

* N = the number of OPL samples.

Table S2. All measurements from the left and right optic lobes are monotonically related*.

	Optic lobe area	Medulla area	Tangential zone area	Cell islands area	Cortex thickness	Radial column zone thickness
Spearman's correlation coefficient	0.991	0.984	0.984	0.965	0.949	0.958
p value	< 0.001	< 0.001	< 0.001	< 0.001	< 0.001	< 0.001

*This suggests that the developmental pace of each pair of optic lobes is similar.

Table S3. Standard errors not shown in Figure 4E and 4F.

Stage	Cortex (%)	R.z (%)	T.z. (%)	Islands (%)
st. 22	20.93±2.69	0.00±0.00	79.07±2.69	98.19±0.26
st. 23	16.13±1.09	0.00±0.00	83.87±1.09	92.24±0.74
st. 24	13.78±0.71	11.51±1.57	74.72±1.50	80.06±1.49
st. 25	12.34±1.03	14.15±0.33	73.51±0.83	79.05±0.95
st. 26	13.90±0.96	13.02±1.82	73.08±1.62	80.81±1.88
st. 27	13.92±0.66	14.93±1.12	71.14±0.76	72.72±1.00
st. 28	14.86±0.62	14.47±0.68	70.67±0.88	77.12±2.41
st. 29	15.48±0.70	17.70±1.12	66.82±0.57	71.23±2.81
hatchling	18.95±0.72	20.41±2.06	60.63±2.77	67.47±3.89

Table S4. Standard errors not shown in Figure 6E and 6F.

ML (cm)	Cortex (%)	R.z. (%)	T.z. (%)	Islands (%)
0.9	16.22±1.64	17.04±1.21	66.73±0.77	61.38±2.33
1.0	17.18±1.19	16.27±0.83	66.55±1.46	56.40±1.65
1.1	15.95±0.83	16.54±0.77	67.51±0.86	59.06±1.31
1.3	17.11±1.51	15.01±0.82	67.88±1.83	55.11±2.96
2.5	15.94±0.60	23.66±1.51	60.4±2.10	47.44±0.68
2.7	16.33	24.67	59.01	45.96
4.3	15.08±0.25	31.79±3.10	53.14±3.35	45.17±4.9
8.0	14.18±1.02	35.21±0.05	50.61±1.08	37.12±3.54
13.6	11.68	34.24	54.08	37.04
17.0	9.69±1.86	35.58±3.27	54.73±1.41	27.3±0.72
19.5	9.94±0.22	29.91±0.06	60.14±0.16	23.81±1.41
22.5	9.24	30.64	60.11	26.36
30.2	8.52±1.33	30.99±0.09	60.49±1.24	24.99±4.69

Table S5. Standard errors not shown in Figure S1.

Stage	Input (%)	T.z. (%).	Output (%)
embryo	18.80±1.39	72.57±1.38	8.63±1.47
juvenile	20.75±0.60	74.22±0.90	5.03±1.40
adult	22.93±0.75	74.61±0.44	2.46±0.47
